# The Politics of Clinic and Critique in Southern Brazil

**DOI:** 10.1177/02632764221076430

**Published:** 2022-04-01

**Authors:** Dominique P. Béhague

**Affiliations:** Vanderbilt University and King’s College London

**Keywords:** critical pedagogy, decoloniality, epistemology, feminist theory, Foucault, ontology, psyche

## Abstract

Drawing on a historical ethnography of how Brazil’s post-dictatorial psychiatric reforms have shaped young people’s lives, this paper builds on Eve Sedgwick’s analysis of the hermeneutics of suspicion to show that narrow applications of Foucault’s *biopower* concept nurture forms of resistance to bio-reductionism centred primarily on epistemic deconstruction. To unsettle this hermeneutic, I put young people’s theories of power into conversation with Georges Canguilhem’s concept of the *milieu* and with feminist scholars’ work on prefigurative politics. I introduce the concepts of *threading* and *unthreading* to consider how one subject of biopower, the child-like biobehavioural figure, was continuously being *threaded* within a specific milieu and in relation to another key figure: the elite angst-ridden ‘storm-and-stress’ adolescent. Young people’s subsequent *unthreading* and *reweaving* politics, flourishing in co-construction with what I call the politicizing clinic, illustrate how decolonial pedagogies can incrementally change the patterning of social life.

## Introduction

Recently, social theorists have called attention to the limits of overly structuralist and narrow uses of Foucauldian theory. A ‘we know better’ Foucauldian ‘industry’, writes Isabelle Stengers, has taken hold, focused primarily on analyses of biopower, knowledge-production, and regimes of truth (Stengers, 2008, 2019). Emphasizing discourse, text, and semiotics over ‘what people do, how they live, [and] the larger world of the material existence they inhabit’ (Hacking, 1998: 86), this form of Foucauldianism tends to side-step the multidirectional ways knowledge gains traction and is co-constructed (Greco, 2004; Jasanoff, 2004). As Martin Savransky has argued, these problems reflect not Foucault’s scholarship, but the ‘Governmentality Studies’ school created by his followers, who tend to frame subject formation as a ‘linear and direct vector of [clinical] power’, leaving questions of heterogeneity, change, and resistance comparatively unexamined (Savransky, 2014: 104–5).

Governmentality Studies approaches are situated in a broader style of social theorization stemming from the highly influential methods of critique developed by Marx, Nietzsche, and Freud, and coined by Paul Ricoeur as the ‘hermeneutics of suspicion’ (Felski, 2015). The primary aim of suspicious hermeneutics is to ‘unmask’ and ‘deconstruct’ the ways ideologies persuade people to participate, sometimes unknowingly or against their own will, in systems of oppression (Guess, 1981). Eve Sedgwick has argued that a hermeneutic of suspicion adopts a ‘paranoid’ stance that has ‘made it less rather than more possible to unpack the local, contingent relations’ in which knowledge gains epistemic, affective, and material traction (Sedgwick, 2003: 124). Drawing on Sedgwick’s work, Lauren Berlant has urged scholars to move from a politics of ‘denouncement’ to one saturated in ‘the ethical pressure to figure out repair in the face of intensifying world disrepair’ (Berlant, 2019: 4).

This paper takes up Berlant’s invitation by studying different forms of ‘critique’ as socially and historically situated practices (Latour, 2004; Fassin, 2017). How, I ask, are hermeneutic styles and practices deployed, enacted, arranged, and normalized, and with what implications for social, political, and mental life? To explore this question, I draw from a historical ethnography initiated during the large-scale governmental, economic, and institutional reforms that swept through Brazil in the 1990s and 2000s, following the end of the military dictatorship in 1984. In psychiatry and medicine, reformists were inspired by schools of thought that are often emblematic of suspicious hermeneutics: psychoanalysis, Marxist-inspired social medicine, radical anti-psychiatry writers such as Italian Franco Basaglia, Paulo Freire’s critical pedagogy, and Foucauldian theories of governmentality (Tenorio, 2002). Those leading psychiatric reform, being sensitive to the ‘social and economic determinants’ of mental health, instated policies to expand state-funded community-based care and reduce psychiatric hospitalization, and they were explicitly critical of the pharmaceuticalized neuroscience logics that were on the rise in the US and the UK in the 1990s (Conde, 2020).

Though Brazilian psychiatric reform became known internationally as a success, from the outstart many psychiatrists, psychologists, public health officials, social scientists, and scholar-activists worried that reform was only superficially concerned with ‘the social’ (Yasui, 2010). In relation to young people specifically, they cautioned against the expansion of Anglophone psychiatric and economic values in Brazil, and the likely iatrogenic impacts of the rise in diagnoses such as attention deficit hyperactivity disorder and depression. Instead of experiencing socially sensitive therapy, young people were starting to be labelled and medicated. Using a suspicious style of critique, a meta-narrative crystallized: deep analysis of the ideological forces subtly persuading clinicians and patients to embrace biological reductionism would be needed for lasting reform. Like in other countries undergoing psychiatric reform, this narrative served as a call to arms, and it continues to dominate today as public health officials and leading social psychiatrists analyse why their experiments in radical care seem mostly to have failed.

In this paper, I unsettle this well-recited account by thinking with and against a Foucauldian emphasis on the power-knowledge nexus, or as Stengers puts it, by ‘following and betraying’ Foucault (Stengers, 2019: 4). My focal point is not on the rise of biological reductionism, but modes of life that diverted from this then-emerging trend. In the late 1990s, I met several young people who experienced forms of therapy that aligned with reformists’ ideals. Together and sometimes in tension with therapists, teachers, and parents, these young people co-created what I call a ‘politicizing clinic’, a therapeutic space where clinicians and patients challenged the imperative for a ‘cure’, stretched definitions of ‘care’, and democratized the therapeutic encounter (Béhague, 2009; see also Giordano, 2018; Brotherton, 2020). Critique of normative ways of knowing was only one component in the making of the politicizing clinic; the encounter within the four walls of the clinic or school was only a stopping point in much broader, multi-layered, multi-generational, and multi-institutional journeys. My claim is that a full rendering of the politicizing clinic necessitates attention to less visible and less valued hermeneutic approaches, and to how these interact with dominant ‘suspicious’ ones.

I consider the emergence of the politicizing clinic space in three steps. First, I show how Foucauldian theory, when used narrowly by reformists, clinicians, and educators, limited the potential of radical reform by predetermining practices of critique as needing to be centered first and foremost on epistemic deconstruction and resistance. Second, to move beyond a deconstructionist orientation (Hacking, 1999), I put what young people taught me about their theories of power into dialogue with Georges Canguilhem’s well-known argument that ‘the living make their own *milieu*’ (2008: 111). The *milieu* serves as a ‘constant reminder of the self-organizing, dynamic, self-regulating complexity of living systems’ (Rose, 2013: 23); instead of indexing ‘a formation around a centre’, it conjures ‘the representation of an indefinitely extendible line or plane [. . .] a pure system of relations without supports’ (Canguilhem, 2008: 103). By putting the spotlight on the *making* of *relations*, the milieu does not a priori situate the institutionalized episteme at the centre, or ‘resistance’ on the periphery. Milieu-making, I argue, is an apt reflection of how some of my young interlocutors sought to theorize the who, what, where, and how of power formations.

Building on young people’s insights, I explore how one subject of biopower, the developmentally stuck and child-like biobehavioural figure, was continuously being *threaded* into and within a specific milieu and in relation to another key figure: the elite angst-ridden storm-and-stress adolescent steeped in self-made transformation and progress (Baumeister and Tice, 1986). My use of the *threads*/*threading* metaphor expands on the ‘how’ of milieu-making and is inspired by feminist scholars’ work on *prefigurative* ways of analysing systems of oppression; that is, from the vantage point of an imagined *not-yet* (Anzaldúa, 1987). As Elizabeth Povinelli writes, emergent figures of biopower are more than ‘diagnostic’, for they also indicate ‘a possible world beyond or otherwise to their own forms’ (Povinelli, 2017: 57–8).

In a third section, I explore how the politicizing clinic flourished as young people amplified their genealogical, sociological, and prefigurative insights by tugging at the threads – the ideas, values, affects, and routines of living and relating – that bound the figures to their respective *milieux* and to one another. *Unthreading* intersects with Ian Hacking’s ‘reworking’ notion (Hacking, 1999), but it emphasizes the systematic and relational ways young people began to demobilize hierarchies by forming new relationships with figure and milieus, by actively repatterning life itself. Unthreading, a kind of critique-through-experimentation (Meyers, 2022), departs from paranoid critique – and associated analytics, what Eve Tuck has called ‘damage-centred’ research (Tuck, 2009) or what Robbins and others have critiqued as the ‘suffering stranger’ paradigm in anthropology (Robbins, 2013) – by explicitly focusing on incremental mechanisms of change. I conclude by considering how my young interlocutors’ modes of critique reflect decolonial pedagogies and reweaving world-making practices (Lugones, 2003; Escobar, 2018).

Historical ethnographies are well placed to facilitate theorizing potentiality and unthreading politics because they can prioritize on-the-ground observation, comparative analysis over time, and divergences from normative trends. Colleagues and I repeatedly interviewed a group of nearly 100 young people, randomly selected from the 1982 Pelotas epidemiological birth cohort study (Victora et al., 2003) as they grew up during the decades of psychiatric reform, from their teen years (1990s) through their adulthood (2000s onward). We also conducted participant observation in schools and clinics and interviewed over 100 professionals working in a range of institutions. In what follows, I interweave theory with the experiences of two teens, Alex and Beto, selected to exemplify different modes of engaging with the politicizing clinic and critiquing the situated making of the biobehavioural and storm-and-stress figures of biopower.

## Biopower: Bioresistance

Brazilian scholars intensified their engagement with Foucault’s works in the late 1960s and early 1970s, at the height of the military dictatorship, a time when his writings on the panopticon and bodily discipline resonated with Brazilians’ lived experiences of authoritarianism (Conde, 2020; Machado, 2006). Foucault wrote about the dictatorship in a series of lectures delivered in Brazil during these decades, articulating a view of philosophy as a ‘form of radical journalism’ (Rodrigues and Penzim, 2011: 16). Foucauldian analyses were used to show how psychiatric hospitalization, which had swelled during the dictatorship, functioned as a core tool of political repression. After the reinstatement of democracy in 1984 and the constitutional revision of 1988, clinicians and activists turned to Foucauldian-inspired anti-psychiatry writers of the 1970s and 1980s to shed light on how psy-expertise had become an insidious technology of control not just in medicine and psychiatry but also education, the justice system, and public health (Yasui, 2010).

When I began fieldwork in the mid-1990s, broad-brush Foucauldian analyses were pervasive in the social sciences, social medicine, and critical pedagogy (Machado, 2006). Psychiatrists and academics argued that cognitive behavioural psychology, which gained considerable ground in public schools during the dictatorship, had primed teachers, school psychologists, and students to readily accept the biological model of the mind that was starting to become dominant in the US (Barbosa, 2012). Even when students’ *inattention* and *agitation* were framed not as biological conditions but as legitimate responses to under-resourced school environments, school staff worried that such labels stigmatized students and narrowed proposed solutions. Professors in the faculty of education warned against behaviourist epistemologies, and they put Foucault’s biopower concept into dialogue with the then recently unbanned works of Brazilian educator Paulo Freire on critical consciousness (Freire, 2005 [1970]). Listening sessions and student government were set up in schools, and, as one of my psychiatrist interlocutors told me, therapists receiving referrals from schools sought to create ‘socially sensitive’ forms of therapy that might attend to ‘deep-rooted inequities and the scars of the dictatorship’.

These changes provided new opportunities for young people to air their frustrations with education and psychology. Alex, aged 15 when I met him in 1997, was unabashedly vocal about his disgruntlement. ‘I’ve been sent [to the school psychologist] a few times’, he explained. ‘This last time, instead of reprimanding all the kids who were talking, the teacher pegged me. I remained calm but she stormed out, angry like a *fera* (wild animal). She said she was disgusted by my face . . . Then she sent me to the psychologist.’ Alex shifted to the first-person plural to signal the well-known sentiment that kids from ‘poor neighbourhoods’ were far more likely to be unfairly referred for counselling. ‘They target *us*’, he continued. ‘The psychologist is just there to say they have one . . . to make money. She kept asking me about my feelings, my anger, and *impulses*, but I don’t see what that’s got to do with it. I thought she might explain why the teacher hates me.’ Facilitated by the broader diffusion of grass-roots politics and critical theory in the post-dictatorial era, contestations such as Alex’s became more common and public.

Clinicians, in turn, debated the complex social causes of mental distress with growing fervour. Encouraged in part by the rise of young people’s voices, they relied on psychoanalytic approaches to strengthen their clients’ capacity to critique the social causes of their distress and emotional struggles. Imparting critical faculties included encouraging young people to ‘the face of large economic and structural forces biological epistemologies and the way they obfuscate the social – what some psychiatrists, pedagogic specialists, and professors identified as Foucauldian biopower.

The emphasis on deconstruction was on one level curious, since a biological episteme that excluded the social was at the time not dominant. Social medicine and psychoanalytic epistemologies were far more mainstream in Brazil than in the US or the UK, and categorical diagnoses found in the American Psychiatric Association’s Diagnostic and Statistical Manual of Mental Disorders were explicitly not used in clinics. When clinicians suggested prescriptions, which many relied on as a last resort, they explained that medication use was a temporary facilitator for the more important work of deep analysis (Freitas and Amarante, 2017).

In my estimation, clinicians’ emphasis on a soon-to-be imported (Anglophone) ‘bio’ was emblematic of the popularity of deconstructionist social theory (Hacking, 1999); it was pre-emptive of a presumed likely future, a form of resistance that attenuated their sense of paralysis in the face of large economic and structural forces affecting young people’s lives. Clinicians’ commitment to bio-epistemic deconstruction resonates with Sedgwick’s argument that ‘suspicious’ hermeneutics are comforting in the ‘reframing’ and ‘demystifying’ work that they prescribe (Sedgwick, 2003). Yet, while promising a significant undoing, such suspicious analytics often resulted in what Ian Hacking has called the ‘primacy of verbalism’ (Hacking, 1998: 86) – a thin use of critical theory that provided practitioners with an epistemic locus of intervention, a presumed effective pathway to addressing the social, economic, moral, and institutionalized dynamics that Alex and others brought to the table.

Narrow uses of critical theory also reinforced my interlocutors’ reticence to interrogate the deeper eugenicist history of biological epistemologies in early 20th-century Freudianism in Brazil (Costa, 1976; Facchinetti and de Castro, 2015, see also, Fitzgerald and Callard, 2015). Among other major schools of thought, psychoanalysis grew out of 19th-century progressivist values and evolutionary theory in which a core developmental assumption operated: the mind of the child was to be studied as a window into the mind of ‘inferior’ classes of people: women, non-whites, the poor, and those with mental illness or disabilities. This comparison, so vital to colonial institutions and practices, juxtaposed ‘upper-class intellectualism’ and the ‘superior’ developmental capacities of elite children against the ‘inborn’ and ‘retrograde psyche’ of a racialized ‘underclass’, found in the ‘stagnated development’ of black, brown, and poor children and ‘child-like’ adults (Bowler, 2003). Throughout the 20th century, Brazil’s two-tiered psychiatric system – psychoanalysis for the rich and hospitalization for the poor – was entwined with a whole range of institutionalized power hierarchies that used biological logics to justify subjugating whole populations (Duarte, 1999–2000).

Reflecting on this history, one psychiatrist I interviewed insightfully (if atypically) suggested that young people such as Alex were struggling against a priori assumptions of their pedagogic and psychological capacities that were deeply embedded in the 19th-century figure of the degenerate child (and adult). This figure’s ‘stagnated development’, he continued, has been perennially compared to the ‘advanced development’ of the elite storm-and-stress adolescent figure, a figure that owes much to the work of psychoanalyst Stanley Hall at the turn of the 20th century. Though my interlocutor referenced the four ‘figures’ central to Foucault’s biopower (Foucault, 1981), like Povinelli, he considered the late 20th-century biobehavioural figure to be an opener for contestation, more than just diagnostic of the weight of the past.

My fieldwork indicates that languages, values, and aesthetics linked to these two figures were being used and distributed in ways that brought history into a palpable and manipulable present. In the ethnographic and epidemiological studies of the Pelotas cohort, we found that young people were more likely to be framed (and to frame themselves) in behavioural terms – as agitated, inattentive, and with disordered conduct – if they were young men, reported lower family income, and if they identified as ‘black’ or of ‘mixed’ race (Béhague, 2015). In contrast, a framing reminiscent of a psychoanalytic ‘storm-and-stress’ view of adolescence was more frequently used for and referenced by young women, and those who reported higher family incomes and identified as ‘white’.

The circulation and internalization of the biobehavioural figure tended to intensify with chronic medication use, which in turn fed into an immutable biological theory of cause, usually in young people’s adult years, in the latter 2000s. As an older teen, Alex was referred to a psychiatrist through his local clinic where he sought help for persistent insomnia, headaches, and a complete lack of energy. After a year of talk therapy, the psychiatrist prescribed an anti-depressant somewhat begrudgingly as a last resort. Over the subsequent years, Alex tried unsuccessfully to wean himself off his medication and eventually decided his intractable ‘depression’ would need medication for a long time, if not indefinitely. I witnessed this clinical trajectory time and again.

Many psychiatrists viewed such outcomes as indications of the clinician’s ‘failure’. It was at these moments that the limits of suspicious hermeneutics were most glaring. Psychiatrists often analysed cases such as Alex’s in relation to the intractable force of poverty and the insidious persuasions of a latent bio-neuro episteme, what some called, drawing on Marxist theory, ‘the pharmaceuticalization of poverty’. Sometimes, clinicians moved beyond this interpretation by situating pharmaceuticalization within more nuanced histories of governance, political disenfranchisement, dictatorship, and even enduring structures of colonialism. Yet their overwhelming sense of disempowerment often prompted a return to suspicious hermeneutics centred on resisting epistemic reductionism. While engendering a sense of agency, this also fed what I have described elsewhere as a recursive gridlock of biopolitical ‘reproduction’ and bio-epistemic ‘resistance’ (Béhague, 2015).

One way that clinicians and young people began to break from this gridlock was to consider how incremental departures from bifurcating trends might take shape. Tuck has described this as a process of ‘thirding’, a refusal of the either/or ‘reproduction-resistance’ binary (Tuck, 2009). Beto’s life exemplified one such departure. When I met him as a young teen, his struggles mirrored Alex’s; he was at risk of failing fifth grade, had failed two prior grades, and was constantly getting into trouble. ‘They can’t write on my card that I’m aggressive’, Beto baulked after the school psychologist offered anger-management strategies. ‘I admit it. With teachers, if they yell at me, I find it hard to keep still. The blood rises to my head . . . I’m better now, but shit, some teachers leave you [feeling] completely crazy.’ While Alex’s subsequent return to therapy as a young adult, in the mid-2000s, led to a diagnosis of depression and prolonged antidepressant use, Beto’s return avoided categorical diagnosis and medication use, focusing instead on nurturing the political and social energy needed to incrementally rework the world around him.

## Threads: Threading

The politicizing therapeutic that Beto co-created over those years, both inside and outside of the clinic, while not commonplace was shared by a small but bourgeoning collective of young people. Yet the relative absence of conceptual space for theorizing the politicizing clinic narrowed the transformational role it could have played in Brazil’s post-dictatorial reforms. My claim is that the creation – and potential revival – of the politicizing clinic depends on a non-suspicious theory of power, one that, taking a cue from Beto, asks how the biobehavioural and storm-and-stress figures were continuously being rethreaded by a range of actors within specific material, affective, and semiotic *milieux.*

Foucault drew from Canguilhem’s concept of the milieu to theorise how governing happens through ‘actions at a distance of one body from another’ via the ‘arranging of things’ (Foucault, 2007, in Lemke, 2015: 9). The milieu avoids imputing causality when considering the relationship between objects (diagnosis), subjects (patients), and locations (clinic) (Caduff, 2019); it helps decentre facile ‘Foucauldian’ analyses that overemphasize how scientific and clinical epistemes shape subjectivity in the clinic (and school). If, as Barad (2007: 236) has argued, ‘causal relations do not pre-exist but are rather inter-actively produced’, then more detailed attention to milieu- and figure-making, to the threading practices that do the arranging, can provide glimmers for *enacting* an otherwise.

To demonstrate the breadth of the threads that tied the biobehavioural figure to its milieu, outside the clinic proper, let me start with everyday life in the shantytown, or *vila*, the term preferred by most residents. Parents living in the *vila* were intensely divided on whether young people should be given the freedom to roam the streets. Many favoured exposing youth to the fullness of both *vila* life and ‘better’ neighbourhoods even if, *and because*, this meant intensified exposure to conflict and *preconceitos* (prejudices). Parents explained that this conferred social skills, built resilience, legitimized young people’s anger, and fostered pride in their ‘working-class’ backgrounds. In contrast, other parents, including Alex’s mother, Antônia, and Beto’s mother, Celia, believed children should be sheltered indoors. They worried about the dangers that infused the *vila*: violence, police crackdowns, drug trafficking, and what Antônia described as ‘capitalist corruptions and the fast pace of modern life’. The home, they explained, was a place of controlled and effective discipline.

The cultivation of a disciplinarian and sheltered household can be considered a re-threading of the forms of governance that gave rise to psy-developmental expertise during industrial capitalism and colonialism of the 19th century (Castañeda, 2002). Generalized notions of parenting in Brazil have long approached children living in precarious situations as incapable of the psychologically ‘complex’ childhoods of the elite, and as Donna Goldstein has argued, these values can be traced to early 20th-century psychoanalysis and 19th-century evolutionary theory (Goldstein, 1998). In my fieldwork, I found these assumptions embedded in totalizing and semi-totalizing institutions, such as hospitals, juvenile detention centres, and religious and secular residence homes for ‘street youth’. Psychiatrists, psychologists, and social workers employed by these institutions were far more likely than those in out-patient clinics to use racialized culture-of-poverty logics that mixed evolutionary ideas of development with a psychoanalytic emphasis on ‘instincts’. They told me, for instance, how parental neglect, weak mother-child bonding, and over-crowding, all driven by extreme poverty, accounted for young people’s impulsivities and weak ego-formation. In their view, institutionalized behavioural regulation, posited as a parental surrogate of sorts, was the only hope for ‘alienated’ youth.

The assumption that children living in poverty are psychologically incapable of ‘good development’ was woven into the intimacies of parents’ intense disciplinarian practices. Poignantly at the centre of these practices were mothers’ own experiences with psychiatric hospitalization. Antônia and Celia told me of their enduring struggles with ‘nerves attacks’ for which they had both been hospitalized in the 1970s. After distressing experiences in hospital, they became wary of medicine but continued to seek care in no small part because they had become dependent on benzodiazepines and were convinced of the chronicity of their conditions. Yet they parented in ways that supported the very principles used by experts to justify institutionalization. Focusing on breaking ‘bad habits’ through behavioural regulation, including routines of punishment and reward, they were sceptical of the ‘modern’ idea of adolescence – a prolonged and experimental phase of self-exploration – that young people were encountering in school.

Celia, for instance, explained that adolescence, an inappropriate postponement of adulthood, was coddling and a threat to her sons’ budding masculinity. Similarly, Antônia explained, ‘Alex is infantile. He does not want to face up to the reality of adulthood. What he needs is discipline.’ In some families, fathers’ experiences with military service during the time of the dictatorship reinforced parenting practices centred on behavioural discipline. That several of Beto and Alex’s older friends ended up in juvenile detention centres only added to parents’ convictions that strict childrearing was vital. Thus, Alex and Beto entered school as young children already bearing the multigenerational weight of institutionalized and pathologizing *milieu*-making. What fed the biobehavioural figure they started embodying in childhood was far more than a precarious life, conflict in school, or the conceptual reductionism of biobehaviourist framings.

Intergenerationally sedimented routines and perspectives are hard to shake. As Alex and Beto entered their later teen years, they found themselves threading the milieu of the biobehavioural figure ever more tightly. One stark example took shape as they neared their 18th birthdays, the age of enlisting for compulsory one-year military service. Instead of finding a way to avoid conscription, the norm for many middle-class and upwardly-mobile families, Alex and Beto began to imagine, albeit with considerable ambiguity, how ‘behavioural training in the barracks’ might fix their impulsivities and ‘anger issues’ without requiring the emasculating routines of schooling and talk-therapy. While some young people sought social and psychological refuge in service, others, seeking a space of freedom and disruption away from both home and institution, began experimenting with the space of the street. Beto was desperate to leave his home – his mother, he said, ‘ran family life as if in the time of slavery’ – and while he considered joining the army, he soon realized service would become ‘just another form of imprisonment’. Like Beto, Alex too began hanging out on the street, playing soccer, skateboarding, or just talking and ‘doing nothing’.

‘Taking to the streets’, as young people called it, was by no means straightforward, for it entailed sometimes going against their parents’ wishes and intermingling with the most marginalized and racialized of *vila* youth. Both Alex and Beto were light-skinned and variously identified as either white or brown, depending on context. Given the fluidity of Brazilian racial politics, where the cues of blackness are co-constituted with material aspects of class (Guimarães, 2006), in the space of the street, they were more apt to be seen as black and directly discriminated against by police, outreach workers, and neighbours. Though spending time on the streets saddled Alex and Beto with the poor, racialized, and behaviourally disturbed figure, it also gave them access to grass-roots politics and to a vibrant space of collectivizing disobedience. This was especially the case in *vilas* where a politics of solidarity, and what James Holston has described as ‘insurgent citizenship’, had swelled in the lead-up to the fall of the dictatorship (Holston, 2008). In time, Alex and Beto began articulating an explicit commitment to what they identified as Afro-Brazilian culture and politics, and to *not* seeking shelter from the intensified discrimination that street life entailed.

Street politics also encouraged some young people to vociferously challenge the psychologized targeting of students in school. Young people’s critique of the school-psy space gained credibility as they encountered doctors and psychiatrists who spent time in the *vila*. When Alex and his skateboarding friends began advocating for the installation of an official skateboard park in the city, they were noticed by a community psychiatrist, Jorge, who spent time conducting home visits in the *vila* near the primary care clinic where he worked. Jorge was involved in supporting local activists as they fought with city officials for better living conditions, and he played a key role in validating Alex’s budding activism.

Less visible but no less important were the horizontal forms of politics that Alex and Beto nurtured as they pushed for more outward forms of living and in so doing, experienced intensified conflicts with childhood friends, particularly those who had become ‘good students’ and whose parents intentionally kept them from socializing with youth in the *vila* whom they considered *marginais* (marginals). ‘Good students’ also used school-based therapy, but in a starkly different way: to grapple with the anxieties of school demands and the solitude that surfaced as their parents imposed social distance. Cultivating a psychoanalytic storm-and-stress adolescent subject-form, they contrasted their psychological ‘hard work’ and ‘capacities’ with the ‘agitations’ and ‘addictions’ they witnessed unfolding among their ‘*marginal* peers’. These two figures – the child-like marginal and the upwardly mobile storm-and-stress adolescent – were deeply embroiled in a micro-politics of belonging and exclusion, with tensions running so high that Alex and Beto sometimes spoke about feeling more distressingly othered by friends and neighbours than by teachers and psychologists.

It was at this juncture that Beto’s politics began to differ from Alex’s. Alex moved towards practices that hardened divisions, joining friend groups that publicly committed to maintaining social boundaries. In contrast, Beto blurred social divisions and spoke avidly about ‘learning how to get along with everyone’. He moved through multiple social and geographical spaces, maintaining his street life, actively befriending local drug dealers while remaining in school and befriending peers who were slightly ‘better off’. He recalled softly confronting the grandmothers in the *vila* who were known for yelling ‘*Malandro*!’ (vagabond) or at times ‘*Mandingo*!’ (person of African descent) from their homes through open windows as ‘street-wise’ teens passed by. While Beto was criticized by some of his friends for not ardently confronting bigotry, he explained that aligning *only* with street politics or *only* school life divided people. He worried that Alex’s form of politics was too exclusive and that it played into the hands of those who blamed ‘poverty’ – instead of the hierarchies that reproduce segregation and discrimination – for social and psychological ills.

Some scholars might agree with Beto. Paulo Freire famously cautioned that certain forms of activism can become sectarian and anti-dialogical (Freire, 2005 [1970]). Teresa Caldeira has described how urban street politics are powerful if also paradoxical in that they celebrate alterity while also fracturing public spaces by deepening gender hierarchies (Caldeira, 2012). Dreyfus and Rabinow (1982: 169) suggest that for Foucault, ‘repression itself is not the most general form of [modern] domination . . . [Rather,] it is the belief that one is resisting repression, whether by self-knowledge or by speaking the truth, that supports domination, for it hides the real working of power.’ Thus, in one interpretation, the suspicious deconstructionist hermeneutic that Alex employed can be considered a thread among others that tied the biobehavioural figure to its milieu. Yet neither Freire nor Caldeira, nor perhaps Beto, would suggest that fervently confronting normativity only feeds oppressive structures via the well-worn path of false consciousness (Theodossopoulos, 2014; Sloterdijk, 1988). Critiquing authoritative knowledge constituted a productive space of debate for both Beto and Alex.

At the same time, Beto’s positionality suggests that political practices arising from suspicious hermeneutics are incomplete. Alex’s form of politics was more intensely anti-classist, leaving intersections with race, racism, gender, and sexuality comparatively intact. Beto’s commitment to the affective and relational details of world-making echoes scholars’ calls for a consideration of the limits of ‘resistance’ modalities and rights-based recognition politics (Boland, 2013; Simpson, 2017). For Sedgwick, suspicious hermeneutics have become a ‘mandatory injunction’, entirely ‘coextensive with critical theoretical inquiry’ rather than one among other possibilities (Sedgwick, 2003: 125–6). If as Rita Felski has argued, a hermeneutic of suspicion is steeped in a spirit of disenchantment and negative affect (Felski, 2015), what other affective moods are possible (Stewart, 2007)? How do Beto’s politics suggest other possible hermeneutics, particularly those emanating from decolonial world-making pedagogies (Singh, 2019) that grow out of the unthreading of the figures-*milieux* constellation?

## Unthreading

Beto seemed less interested than Alex in providing his friends (or me) with a clear picture of his convictions, critiques, or life choices. He moved through life more as a para-ethnographer (Holmes and Marcus, 2008), intermixing his observations and analyses of the patterning of social life and the threading of the milieu with its incremental unravelling. It was within the continual act of observing and sometimes participating in the rethreading of the figure to its milieu that the possibility of unthreading began, not just via critique of powerful ways of knowing, but through practising an *otherwise* (Povinelli, 2016).

As Beto circulated through school, street, and other neighbourhoods, encountering people from all walks of life, he challenged his parents’ disciplinarian position that ‘adolescent freedom’ led only to fragility and instead pushed for his own unique form of adolescence embedded in a wider geo-moral-spatial way of living. In so doing, he began to embody *both* the ‘feminized’ middle-class figure of the ‘good school-going’ adolescent *and* the ‘masculine’ figure of the behaviourally-disturbed student. As he did this, he incrementally weakened the many binaries – school/street; female/male; upwardly mobile/working-class; dark-skinned/light-skinned – through which the biobehavioural and storm-and-stress figures have long been (and continue to be) created. Beto’s form of politics thus went beyond resisting normative prototypes by calling out and deconstructing psy-languages, for he embodied portions of the figures, moving through space and relations in new ways.

While Beto was in the minority, his way of life and values were becoming an identifiable orientation among a small collective of young people. Like him, I witnessed other young people use their adolescent transitions to weaken social divisions and subtly unravel social hierarchies, even as they encountered intense criticism from onlookers who laid claim to the moral superiority of this or that way of life. Most young people who encountered such criticisms tended to withdraw into a more sheltered and exclusive form of life. For instance, after much critique and conflict, Alex dropped out of school and adopted a moral-social position that reaffirmed his convictions relating to class struggles, his masculinity, the futility of education, and the legitimacy of street life. This brought him a deeper sense of belonging and some respite from chronic discrimination. It was also strategic: Alex knew discriminatory hiring practices were such that employment was by no means assured even for those who managed to survive and complete secondary school.

To my mind, the key question was not why Alex left school, as educators and psychologists so often asked, but rather why others, like Beto, remained. One answer is that for this growing collective of which Beto was a part, the school represented more than a pathway to employment. School was a budding space of politics. Beto was curious about the opportunities for self-determination that had been spurred by pedagogic reform. Following ideals of critical pedagogy, teaching methods turned towards projects-based learning, student councils were formed, elected student-leaders conducted student-feedback surveys, and psychologists helped teachers shift away from punitive disciplinary practices. Many teachers pushed back, claiming their authority was being undermined, while others rallied to support what they identified as the democratic reconfiguration of learning (Barbosa, 2012). Most low-income youth were intensely sceptical of school reforms, and they criticized peers for supporting what they considered mere pro-democracy lip-service. They were fully aware that many teachers considered ‘kids from the slums’ to be ‘developmentally stuck’ and ‘unintelligent’. Scepticism surrounding education was also fuelled by families’ multiple experiences with the abuses of totalizing institutions. Both Alex’s and Beto’s mother had painful memories of their time in the psychiatric hospital and Beto had friends who had been unjustly taken to the juvenile prison in Porto Alegre, the state’s capital.

Yet for Beto, school was a space of politics because within it he could observe the detailed dynamics that fed assumptions of his presumed developmental stagnation. Relative to the totalizing spaces his family and friends had experienced, school was more accessible and malleable while still being cut from the same cloth of institutionalized power. In school, he gained access to – and began to unthread – some of the gendered, classed, and racialized logics and practices that maintained the biobehavioural and storm-and-stress figures. In his 16th year, he decided to run for student council, even though it was filled with ‘better off’ students and he knew he would be ridiculed. Much to his surprise, he won.

Beto was not the only young person I met from the *vila* who entered the contested terrain of student politics. As young people’s sense of agency and moral worth increased, so did their encounters with conflict and denigration. If unthreading practices put strain on the figures, so too did such practices strain those doing the unravelling. Deeply exhausted, after a few months of being on the student council, Beto reconsidered his desire for therapy and returned to the school psychologist. Reminding her of the times he had been sent to her office for misbehaviour, he asked if perhaps his frustrations grew not from ‘adolescent bursts of anger’ but his conflicted relationships with teachers. The psychologist, influenced by social medicine and critical psychiatry, responded by empathizing with the struggles of low-income families. Beto was thankful but uninterested. The psychologist’s framing, though ‘social’, exemplified a ‘damage-centric’ orientation (Tuck, 2009); it was pacifying, especially when compared to his work on the student council where ‘anger’ was more readily understood not as poverty-induced but as worthy, even productive.

A year later, as a 17-year-old, Beto returned to the clinic, this time to an out-patient public sector psychiatrist whom he was referred to through his local clinic. ‘I told him [the psychiatrist], I know that I can easily explode’, Beto said, ‘all those fights I have gotten into, bah, I get into such a *state of nerves*, I know that I can be unbearable to be around. But I had to start working young, after my dad died, and I had to care for my mom . . . I explained *to him*, what really makes me explode is when I am unjustly judged, like what happened at work, when they accused me of stealing.’

Debating the causes of struggle in the spirit of Freire’s critical consciousness was a vital component of the therapeutic encounter for Beto. But epistemic reframing was merely a step to something more. In the clinic, Beto began practising a new way of relating with the therapist, whom he incrementally repositioned not as ‘therapist’ but as representative of the upper-middle class. The clinic became a semi-formal/semi-public space of protest, and the clinical relationship an intentional reckoning with elitism. Many psychiatrists and psychologists noted the contestations their young *vila* interlocutors brought to them, and while not all were up for the clinical challenges this posed, many were. One psychiatrist explained that the *absence* of outright conflict in the clinic would be worrisome given the intense levels of marginalization that minoritized young people endured. As Beto rehearsed conflict and negotiated his worth in the clinic, so too did he gain the energy and confidence to do the same with his teachers and, eventually, his employer.

Growing more confident in challenging authority, Beto began to grapple with the complexities of grassroots activism (cf. Millar, 2014). To remain in school, run for student council, and visit the psychologist while engaging in street politics constituted an affront to nearly every aspect of Beto’s sense of self: his masculinity, working-class pride, and burgeoning ‘good student’ status. In school, his activism often led him to be shunned either for being from the *vila* or for being ‘snobby’, depending on the position of the person passing judgment. On the street, Beto prodded the sexism he found there as much as he was prodded by his friends to consider his own classist and racist ways. Such prodding was virtually non-existent for young men whose families aspired to upward mobility; most lived an increasingly insular life, waiting for the time they might move to a gated middle-class apartment complex. By Beto’s late teen years, his family had become economically capable of moving to a ‘better’ neighbourhood; yet they decided to remain in the *vila*, defying normative views of the ‘good life’ and befriending other families who did the same.

Beto’s mode of living in the world might best be described as infused in praxis-oriented pedagogy (Freire, 2005 [1970]), oriented as much toward the self as towards close others and institutional interlocutors, such as teachers and therapists. For Freire, critical consciousness cannot simply be taught or acquired. It grows out of a multidirectional and dialogic way of analysing and intervening in everyday life. Decolonial theorists have drawn on Freire, among other scholars, to better theorize change in the real world, and to explore how decolonial pedagogies might undo core modernity/coloniality structures and values (Walsh et al., 2018). As Eve Tuck has argued, ‘damage-centric research’ works with the faulty assumption that documenting and demonstrating harm is enough to ensure accountability and political inclusivity. Beto refused damage-centric framings in ways that went beyond epistemic contestation; in micro-steps and through iterations of observation, analysis, and action, he transformed not just the biobehavioural and storm-and-stress figures, but also the *milieux* within and into which they were constantly being woven. This form of pedagogy did more than unmask ideologies: it weakened the ways in which home, street, institutions, self, relationship, and affect intertwined to sustain the figures. That Beto’s experiments in modifying *more* than his own subject position were increasingly shared practices signals how new forms of relating can unthread the patterning of social life.

Throughout these struggles, Beto did not reject diagnosis as a matter of principle. On the contrary, he welcomed it, not so much because having a clearly defined condition reduced stigma or enabled a collective form of biosociality (Rabinow, 1994). Rather, diagnosis served as proof that he suffered *because of* the strains – and hopes – of his fluid, itinerant, stress-ridden, and convivial forms of relating to others. Beto started his therapeutic journey with diagnostic languages typically used for poor, racialized young men (agitation, inattention, aggression). Over his teen years, his way of living became incrementally interjected with framings that were prototypically coded as feminine, white, and middle class – that of an anxious but also enlightened psyche. In contrast to upwardly mobile youth who made this same diagnostic shift and left the biobehavioural figure wholly behind, Beto kept both figures and symptom-clusters centrally at play, incrementally delinking them from their gendered, classed, racialized, affective, and ethical normativities. What Beto seemed most interested in were the modes of relating that sustained categorization, rather than the content of the categories themselves, a position that resonates with Jasbir Puar’s call to foreground ‘categories such as race, gender, and sexuality as events – as encounters – rather than as entities or attributes of the subject’ (Puar, 2009: 168). Beto’s politics of unthreading was thus less deconstructionist and ‘resistant’ than it was imbued with small-scale ontological, epistemic, and relational shifts.

## Reweaving

Suspicious hermeneutics take shape not merely because of how academics, activists, and everyday people interpret the world, but also because suspicious forms of critique gain traction in real-world epistemic, moral, political, affective, and ontological contingencies. Criticism, Biehl and Locke write, has ‘been naturalized as an act of judgment and indictment – a habit of fault finding . . . in a way that reifies ideologies’ (Biehl and Locke, 2017: 32). Foucault’s *Care of the Self* (1986: 51) has inspired scholars and activists precisely because his return to ancient Greek practices of care – what he describes as the ‘work of oneself on oneself and communication with others [as] . . .. a true social practice’ – orients critique away from reification and towards as-yet-unrealized possibilities (May, 2014). The concern with who we *might* be has arguably been present through all of Foucault’s work, whether periodized as ‘early’ or ‘late’. But in their contingent uses, Foucauldian concepts can over-emphasize *epistemic* mechanisms of oppression, thereby promulgating a limited ‘reframing’ and ‘rethinking’ form of resistance politics.

I am not arguing for a ‘post-critique’ shift but rather for more empirical attention to how diverse critical hermeneutics interact as they are used and enacted. On one level, the more common resistance- and epistemes-oriented politics Alex and Beto practised also facilitated Beto’s subsequent more unique intermittent, mobile, and affective politics. These different modes of critique are interdependent and constitute just two possibilities. Yet theorists’ predominant interest in deconstructionist practices may be dampening other styles of critique, ones that, for example, nurture ‘an ecology of questions’ that bear on the ‘complexity at stake’ and that help people refuse to be ‘preyed upon . . . [by welcoming] the test of being in charge of the question which [turned them] into prey’ (Stengers, 2019: 8–10). Without attention to a wider ecology of questions – what Savransky has theorized as an ‘ecology of perhaps’ (Savransky, 2021) – more subtle analytics and transformations already taking place, such as those Beto engaged, will remain under-theorized and unsupported. Sedgwick, I conjecture, might have considered an ‘ecology of perhaps’ to be a form of ‘reparative’ hermeneutics (Sedgwick, 2003).

Threading, unthreading, and reweaving add a complementary dimension to Sedgwick’s reparative concept and to prefigurative practices more generally. Unthreading could be thought of as a form of political agency informed by close para-ethnographic observation of — *and movement within* — the historical and contemporary hierarchical patterning of social life. Unthreading might provide a response to María Lugones’ (2003) call to bridge the utopic work of imagining alternatives with the realities of thinking and living against the grain of oppression. Canguilhem’s *milieu*, as Beto showed me, is both a theory-generating and world-changing tool. The politicizing clinic emerged as young people analysed, interacted with, unthreaded, and reconstituted the threads and threading practices that situate and bind the biobehavioural and storm-and-stress figures to specific *milieux* and to one another. The unthreading and reweaving politics of the politicizing clinic facilitated theorization in action. As Ramzi Fawaz writes, Sedgwick believed we lack not more theories but ‘the ethical, affective, and political orientations required to make those theories have a palpable [. . .] transformative effect on our daily lives’ (Fawaz, 2019: 10).

It is thus not enough to theorize the biosocial, or to diagnose how ‘the social gets under the skin’. Young people intentionally put the stuff of the world under their skin in order to pull at threads of expertise, affect, and subject-positions: in order to reweave new ways of living. This form of political agency reaches through time and space, making no assumptions about the merits of starting from an ‘alternative’ or ‘resistant’ epistemic position. It takes seriously the partial and unfinished quality of critical debate. Sedgwick insightfully cautioned, ‘That a fully initiated listener could still remain indifferent or inimical, or might have no help to offer, is hardly treated as a possibility’ in suspicious hermeneutics (Sedgwick, 2003: 125–6). Inviting the initiated listener to thread, unthread and reweave emphasizes travel across boundaries (Lugones, 2003). Such an invitation helps resituate the way people *relate* to knowledge, institutions, concepts, habits, affects, and to one another (Strathern, 2020). Beto was not the only young person I met who moved through space, neighbourhood, home, clinic, gendered categories, and classist and racialized relations in unpredictable ways. To borrow from Haraway (2015: 164), these young people were kin-making, asking of others their ‘best emotional, intellectual, artistic, and political creativity, individually and collectively’.

On a collectivizing scale, the position Beto embodied aligns with what philosopher and decolonial writer Walter Mignolo terms critical and dialogic cosmopolitanism (Mignolo, 2000; see also Watson, 2014). This cosmopolitanism operates differently from the way rights- and citizenship-centred projects seek to redress the enduring injustices of colonial techno-capitalist subjugation. Not merely a disposition that individuals acquire, dialogic cosmopolitanism arises when people begin to instigate small-scale infrastructural changes that create new social configurations (Skrbis et al., 2004). Centred only partially on epistemic and ideological struggle, Beto’s form of non-suspicious critique was neither conciliatory nor apologetic, neither reformist nor revolutionary; it was, rather, mobile, contested, convivial, de-polarizing, confrontational, hopeful, and reweaving.
